# Development and Multi-Scale Validation of a Finite Element Football Helmet Model

**DOI:** 10.1007/s10439-019-02345-7

**Published:** 2019-09-13

**Authors:** William Decker, Alex Baker, Xin Ye, Philip Brown, Joel Stitzel, F. Scott Gayzik

**Affiliations:** Biomedical Engineering, Wake Forest University School of Medicine, 575 Patterson Ave, Winston-Salem, NC 27101 USA

**Keywords:** Finite element model, Football helmet model, Computational modeling, Finite element analysis, Validation, Injury biomechanics

## Abstract

Head injury is a growing concern within contact sports, including American football. Computational tools such as finite element (FE) models provide an avenue for researchers to study, and potentially optimize safety tools, such as helmets. The goal of this study was to develop an accurate representative helmet model that could be used in further study of head injury to mitigate the toll of concussions in contact sports. An FE model of a Schutt Air XP Pro football helmet was developed through three major steps: geometry development, material characterization, and model validation. The fully assembled helmet model was fit onto a Hybrid III dummy head–neck model and National Operating Committee on Standards for Athletic Equipment (NOCSAE) head model and validated through a series of 67 representative impacts similar to those experienced by a football player. The kinematic and kinetic response of the model was compared to the response of the physical experiments, which included force, head linear acceleration, head angular velocity, and carriage acceleration. The outputs between the model and the physical tests were quantitatively evaluated using CORelation and Analysis (CORA), amounting to an overall averaged score of 0.76. The model described in this study has been extensively validated and can function as a building block for innovation in player safety.

**Electronic supplementary material:**

The online version of this article (10.1007/s10439-019-02345-7) contains supplementary material, which is available to authorized users.

## Introduction

Sports-related head injury is a growing concern within the US, where the Centers for Disease Control and Prevention estimates that 1.6 million to 3.8 million concussions occur in sports annually.^3^ Most of the public attention regarding this issue has been focused on American football. There are approximately 4.2 million total football participants in the US each year among youth, high school, collegiate, professional, and other organized organizations, which equates to one of the highest participation rates of sports in the US.^13^,^26^ Public attention on football is justified, as it has one of the nation’s highest risks of concussion among sports.^3^,^8^,^9^,^27^,^30^,^39^,^44^ The increased awareness of concussion risk has translated to a greater emphasis on technique (e.g. head-up tackling rather than head-first tackling),^7^ rule changes (e.g. penalties for helmet-to-helmet contact), and engineering research and development focused on protective equipment.^40^,^54^

Helmet safety has improved greatly in the past decade, largely due to more rigorous testing and grading systems, such as the independent Summation of Tests for the Analysis of Risk (STAR) evaluation system,^40^,^42^ the National Operating Committee on Standards for Athletic Equipment (NOCSAE),^35^ and annual testing by the National Football League (NFL).^14^,^37^,^54^ Two forms of STAR equations have been developed: the original Football STAR, which utilized a series of 24 drop tests to correlate peak resultant linear head acceleration to concussion incidence throughout a full season,^40^ and the Hockey STAR, which analyzed both linear and angular head acceleration through a series of 12 pendulum impacts (PIs) to predict concussion incidence.^42^ NOCSAE certification tests helmets through a series of drop tests similar to the original STAR, where linear acceleration must fall below a certain threshold for the helmet to be certified.^35^ The NFL linear impactor helmet test encompasses 24 linear ram impacts to a helmeted Hybrid III (HIII) head–neck, with a neck attached to a sliding table. An injury risk metric (Combined Metric) was developed to rank overall helmet performance, which relates peak rotational head acceleration, peak rotational head velocity, and head injury criterion (HIC) across all 24 test conditions.^14^,^54^

Traditionally, head injury biomechanics research has relied on metrics to determine the likelihood of injury. These injury metrics utilize external measurements including kinematics and kinetics to estimate the internal response of a body.^15^,^23^,^40^,^41^,^45^,^51^,^53^ Computational tools, such as finite element (FE) models, allow researchers to directly analyze the internal complex behavior of a system responsible for injury.^47^,^52^ There are FE models of the human brain,^12^,^22^,^25^,^29^,^31^^–^33,46,55 full body,^10^,^18^,^24^ and anthropomorphic test dummies (ATDs).^36^,^56^ All these models can be used to study injury but in different ways. In the ATD models, simulations can be used to calculate head injury metrics from the motion of the head, like what is done with physical ATD’s. These injury metrics include HIC based on head CG linear acceleration^34^ and brain injury criteria (BrIC) based on head CG rotational velocity,^45^ as well as other more recent studies including diffuse axonal multi-axis general evaluation (DAMAGE).^17^ Human body FE models enable the study of the localized tissue response of vital organs (e.g. strain history within the brain) during an impact, which has been correlated to injury using metrics such as cumulative strain damage measure^2^,^46^ and the universal BrIC (UBrIC).^16^

Along with validated head models and injury prediction tools, there is a need for validated helmet models to more fully exploit modeling and simulation in the study of head injuries in contact sports. Because helmet rating agencies and head injury metrics both rely on head kinematics to determine injury risk, it is important to use kinematics to validate FE models of helmets. Such FE models will provide an avenue for researchers to study, and potentially optimize helmet performance.

FE models for personal protective equipment have been developed such as motorcycle helmets^4^,^43^ and military helmets,^1^,^48^,^58^ however, there is a relative lack of football helmet models available given the scope of their usage.^57^ The goal of this study was to develop an accurate representative helmet model that could be used in further study of head injury and broaden the ability of engineers and public health scientists to mitigate the toll of concussions in contact sports. The particular helmet modeled in this study was the Schutt Air XP Pro. The helmet was developed as part of the NFL Engineering Roadmap and is one of a suite of open source computational helmet models and boundary conditions.^20^

## Materials and Methods

The FE model of a Schutt Air XP Pro football helmet (SEI 789102) was developed through five major steps: geometry extraction and development, FE meshing, material characterization, model assembly, and multi-scale model validation. Five physical Schutt Air XP Pro helmets were acquired for scanning, material analysis, and general assembly reference.

### Scanning and Segmentation

**Figure 1:**
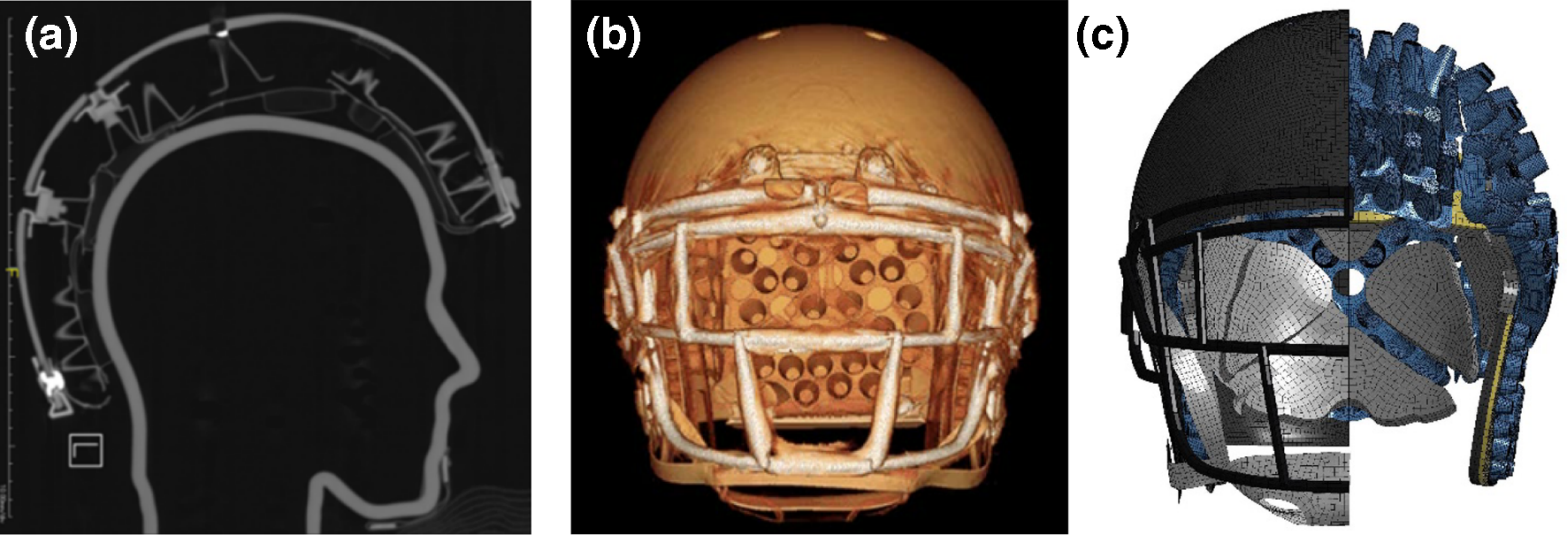
(**a**) Sagittal cross-section of a CT scan donned on a custom 3D printed NOCSAE head form replica; (**b**) rendered view of the CT scan of the full helmet and (**c**) split view of the different components of the helmet mesh.

The geometry of the helmet was developed using various scanning, segmentation, and geometry processing techniques. Computed tomography (CT) scanning was performed on a 64-slice GE VCT scanner (GE Healthcare, Milwaukee, WI). A variety of scans were obtained with the helmet fully assembled, fully disassembled, and fully assembled fit with a 3D-printed NOCSAE head form replica that was free of any metal to avoid artifact (Figure 1). All scans were performed at 0.6 mm cubic voxel resolution. Care was taken to remove and replace any metal components such as screws with nylon counterparts to minimize scanner artifact. Scan data of the fully assembled helmet was segmented part by part using the image segmentation software Mimics (v19.0, Materialise, Leuven, Belgium). The disassembled and fully assembled fit with a 3D head form scan data were utilized throughout the process as supplemental resources for component dimension and helmet fit verification.

### Geometry and Mesh Development

Segmentations were manually cleaned and saved as stereolithography files (.stl). These .stl files were imported and smoothed in Geomagic Studio (2014, 3dsystems, Rock Hill, South Carolina), were fit with CAD geometries if needed in Rhinoceros (v5.0, Robert McNeel and Associates, Seattle, Washington), and meshed using Hypermesh (2016, Altair Engineering, Troy, Michigan). Each part was split along the center sagittal plane in order to retain symmetry during development. A goal of the project was to be able to simulate a helmet impact on a personal computer within a reasonable amount of time. The various components of the helmet were meshed as 1D, 2D, or 3D elements to optimize effectiveness and efficiency of each part. The final mesh for the helmet model is shown in Figure 1. Optimal element size for each component of the helmet was estimated by a simple timestep analysis prior to model development, which analyzed the timestep from a simple shape mesh of various element densities. The minimum allowable timestep was determined to be 5.0E−4 ms in order to target a total helmet runtime of 1 h for a 50 ms simulation.

A midline surface of the helmet shell was obtained from the segmentation data and re-meshed directly in Hypermesh with 2D quad elements. The facemask was created by fitting one-dimensional lines through evenly distributed midpoint measurements along the scan surface, which was then meshed as 1D elements. The two thicknesses of the facemask rails were modeled as two independent parts. The thickness of the facemask was measured at the underlying metal and can be found in the Appendix. The facemask was coated in a rubber material measuring 0.77 mm thick and was not included in the thickness of the facemask parts. This extra thickness was accounted for as a contact thickness. The conical absorbers were meshed with 2D quad elements of edge length 1 mm. The connecting ribs of the conical absorbers were modeled as their own component. Most elements within the model we allocated to the absorbers because they were deemed to have the highest importance in terms of energy absorption. Five different types of foam were found within the padding which were meshed with 3D hexahedral elements. The vinyl coating surrounding the foams was modeled with 2D quad elements along the coincident nodes of the outer surface of the foams. The thickness of the vinyl in the cheek pads was found to be 0.6 mm, where the rest of the vinyl was 0.5 mm and was modeled as such. Connections with the vinyl between foams made up five different pad components (anterior, superior, posterior, and bilateral cheek pads). The chin cup and chin strap were meshed as 2D quad elements with a 1D beam connection of the distal ends of the strap connecting to the helmet shell at the respective locations of the chin strap buttons.

### Material Characterization

Material characterization of the helmet was performed with tensile coupon testing and cylindrical compression testing. Tensile testing was performed across various strain rates of 0.001/s (quasi-static) to upwards of 550/s. Slower rate tests under 100/s were performed on a custom device that allowed displacement-controlled conditions, and dynamic rate tests over 100/s were performed on a custom device that allowed for much higher strain rates with no displacement control. Digital image correlation was used to measure strains for all rates of testing. The tensile testing was performed using ASTM D368 Type V dogbone specimens on the materials shown in Table 1. Compression testing was performed on the helmet shell and foams. All conducted material testing was performed under uniaxial loading. Material characterization of the blue TPU conical absorber was limited due to the lack of adequate thickness required to measure compressive stresses. Instead, material properties of the conical absorber material were back-calculated using single cone FE simulations matched to experiments. These single cones were subjected to two rates of axial compression, with a maximum displacement of approximately 10%, which was held for 10 s prior to unloading, performed on a custom test device (Veryst Engineering, Needham Heights, MA). For the cones, the initial fit response, while true to the data was found to not adequately capture the full component response of the cones within the helmet. Because of this, an extra step was required to tune the material to better capture the response of the cones post-buckling. More on this process can be found in the Results and Discussion. A total of nine material characterization tests were complete and are summarized in Table 1. Note that environmental factors such as temperature and humidity were not considered during model development. Material characterization was performed at Veryst Engineering, Needham Heights, MA.

**Table 1:** Material level validation cases.

Test	Mode and evaluation	Rate(s)	Material model	Experiment
Thermoplastic outer shell	Tension *σ* vs. *ε*	Monotonic: 0.001/s, 100/s, 430/s	Johnson_Cook	
Thermoplastic outer shell	Compression *σ* vs. *ε*	Monotonic: 0.1/s, cyclic: 0.1/s	Johnson_Cook	
Conical absorber	Compression A *F* vs. *D*	Monotonic: 0.1, 50 mm/s	Viscoelastic	
Vinyl foam covering	Tension *σ* vs. *ε*	Monotonic: 0.05/s, 400/s	Fabric	
Black Foam	Compression *σ* vs. *ε*	Monotonic: 0.1/s, 430/s	Fu_Chang_Foam	
High-density grey foam	Compression *σ* vs. *ε*	Monotonic: 0.1/s, 600/s	Fu_Chang_Foam	
Low-density grey foam	Compression *σ* vs. *ε*	Monotonic: 0.1/s, 550/s	Fu_Chang_Foam	
Yellow Foam 1	Compression *σ* vs. *ε*	Monotonic: 0.1/s, 650/s	Fu_Chang_Foam	
Yellow Foam 2	Compression *σ* vs. *ε*	Monotonic: 0.1/s, 600/s	Fu_Chang_Foam	
Chinstrap	Tension *σ* vs. *ε*	Monotonic: 0.0005/s	Elastic	

*σ* Engineering stress, *ε* engineering strain, *F* force, *D* displacement

Material models were created for each component using MCalibrate (Veryst Engineering, Needham Heights, MA) by matching single-element LS-DYNA (Livermore Software Technology Corporation, Livermore, CA) simulations to experimental results. Resulting material models minimized the normalized mean absolute difference between the simulation and the experimental evaluation criteria. Material models utilized in the helmet model can be found in Table 1, with the full validation data in the included Appendix. Complete details of these materials can be found within the model itself (see download location in the Acknowledgments).

The mass of the assembled helmet model was compared on a component level to the physical helmet measurements, with the total mass of the model within 2.5% of the physical helmet. This comparison, as well as the thicknesses of each model component, is shown in the Appendix. The model was developed and tested with specific time step targets for explicit time integration. To accomplish this, mass scaling was performed to a time step of 0.5 *μ*s, which resulted in less than 5% added mass of the helmet. Without mass scaling, the time step of the model is 0.18 *μ*s.

### Helmet Assembly

The assembly of the physical helmet used a variety of fixation methods including screws, brackets, buttons, and Velcro. In order to replicate these in the FE model, we used a variety of modeling techniques including constrained nodal rigid bodies (CNRBs), tied contacts, and surface contacts. The facemask was attached to the helmet shell with CNRBs where physical plastic connections were present (anterior and lateral). An automatic beam to surface contact was also utilized here for severe impacts where the facemask comes in contact with the helmet shell. Within the physical helmet, the conical absorbers were attached to the helmet shell by screws, buttons, and Velcro attachments. Screw and button attachments were replicated within the model with CNRBs, while Velcro attachments were modeled as tied contacts. The padding components of the physical helmet were attached with Velcro to the conical absorbers, which were modeled with tied contacts within the model. Automatic single surface contacts were utilized for contact with the helmet shell, conical absorbers, and padding.

**Figure 2:**
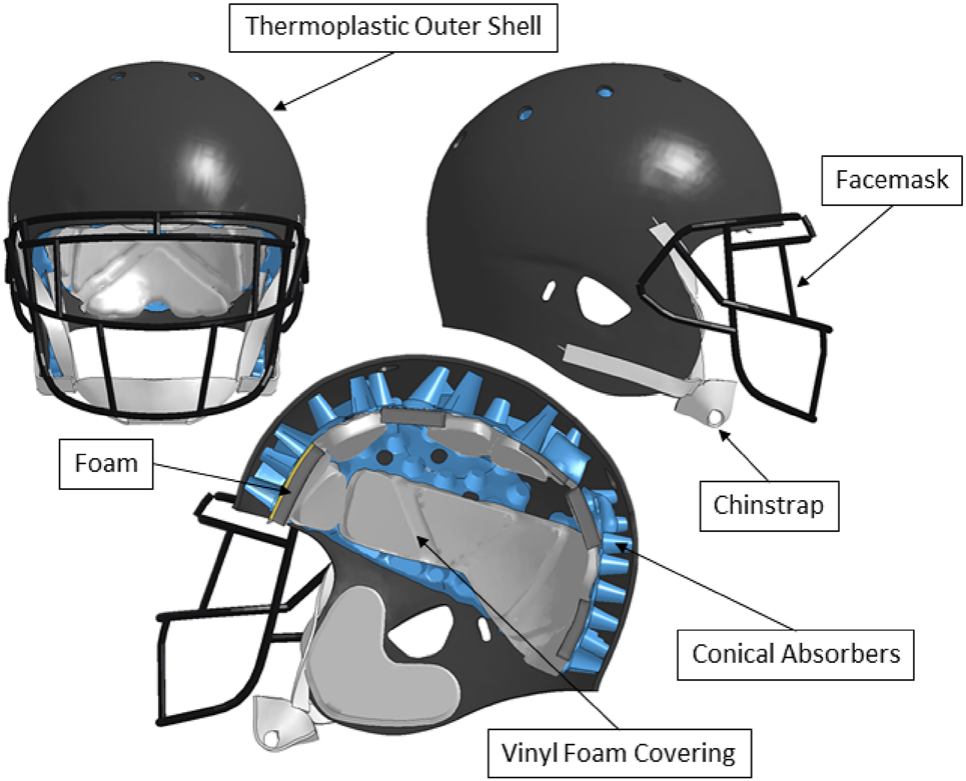
Assembled helmet model front view (left), side view (right), and sagittal cross-section view (bottom).

The padding components within the physical helmet encompassed airflow systems that were categorized as open or closed air systems. The open systems contain air vents on the inferior portions of the vinyl which allow air to exit the system during compression, whereas, the closed systems are fully enclosed vinyl and can be inflated with a pump for fitting purposes. Numerical airbags were implemented to represent the air component of these padding systems. These airbags provide a control volume approach in relating pressure and volume. The venting of the pad systems was defined as a cross-sectional area, which was optimized to match component level pad response (defined in Sub-Assembly Component Testing). The fully assembled helmet can be seen in Figure 2.

### Sub-Assembly Component Testing

A series of component level validation tests were performed at the sub-assembly level of the helmet. These tests include the previously described single cone compression, full padding compression, helmet shell compression, and facemask compression (Table 2). Quasi-static and dynamic compression testing was performed on both open and closed padding systems at 1 and 100 mm/s. The bottom plate of the testing setup was designed to allow to airflow to exit the vent openings of the open system. The closed pad system was tested at 0 psi. Quasi-static testing of the helmet shell and facemask was performed with an MTS Landmark Servohydraulic Test System at a speed of 0.1 mm/s. The helmet shell was compressed in three directions: anterior–posterior, superior–inferior, and lateral, while the facemask was compressed in the anterior–posterior and lateral directions. Each component level test was replicated through simulation and compared to the experimental force–deflection data.

**Table 2:** Various component level testing setups.

Test	Mode and evaluation	Rate(s)	Simulation	Experiment
Meso-scale foam	Compression *F* vs. *D*	1, 100 mm/s		
Helmet thermoplastic shell	Compression (A–P, S–I, lateral) *F* vs. *D*	0.1 mm/s		
Facemask	Compression (A–P, lateral) *F* vs. *D*	0.1 mm/s		

*QS* quasi-static, *Dyn* dynamic, *F* force, *D* displacement, *A* anterior, *P* posterior, *S* superior, *I* inferior

### Helmet Donning

The helmet model was donned on an FE model of a HIII head and neck and NOCSAE head form,^20^ which was then used for the respective impact test matrix shown in Table 3. The pre-fit helmet was first positioned on each head form using a variety of measurements including nose to helmet distance, despite mesh overlap of the components. Once in the desired position, the head form was uniformly scaled down until no longer intersecting with the helmet and gradually expanded through simulation to the original shape using the *boundary prescribed final geometry* function in LS-DYNA. Mid-plane nodes of the helmet model were constrained laterally and vertically to keep the helmet in place during the fitting simulation and to allow natural deformation. The process of fitting the helmet onto a head form can compress the foam components to a high degree. The stress of the deformed foam components was retained for further simulation using the initial foam reference geometry. This uses the pre-fit nodal locations of the foam to calculate the stress of the foams at the initial state of the simulation. The chinstrap was fit onto the NOCSAE and HIII head forms prior to the validation simulations. The chinstrap was defined in contact with the helmet shell, but not the cheek pads, in order to simplify this system and maximize stability.

**Table 3:** Summary of the impact conditions and outputs used for helmet validation.

Impact condition	Dummy head form	Impact location	Impact velocity (m/s)	Outputs
Pendulum impact	HIII	Back, front, front boss, side	3.0, 4.6, 6.1	CA(X), HLA(XYZ), HAV(XYZ)
Linear impactor	HIII	A, AP, B, C, D, F, R, UT	5.5, 7.4, 9.3	*F*(XYZ), CA(X), HLA(XYZ), HAV(XYZ)
Drop impact	HIII	Back, front, side, top	2.9, 4.9, 6.0	*F*(XZ), CA(Z), HLA(XZ)
Drop impact	NOCSAE	Back, front, mask, side, top	2.9, 3.7, 4.9, 6.0	*F*(XZ), CA(Z), HLA(XZ)

*F* force, *CA* carriage acceleration, *HLA* head linear acceleration, *HAV* head angular velocity

### Validation Method

**Figure 3:**
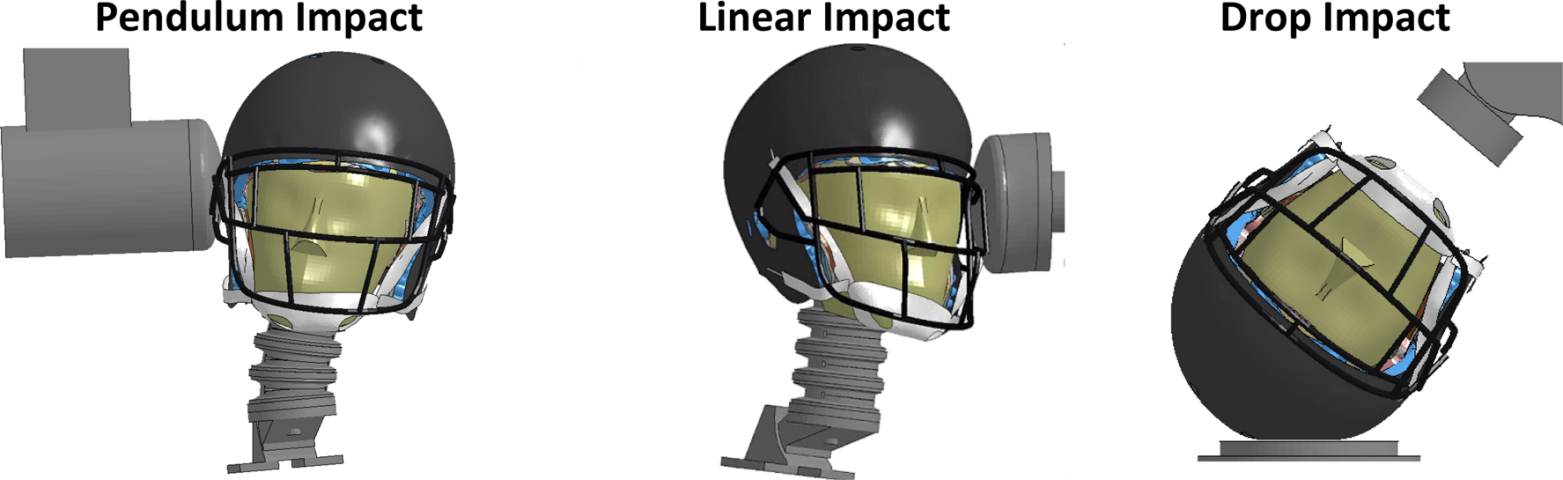
Visual overview of the three impact conditions used for helmet validation. HIII head form is shown.

All simulations were run in LS-DYNA (v7.1.2)^28^ on the Distributed Environment for Academic Computing (DEAC) high-performance computational cluster at Wake Forest University. The fully assembled helmet model was validated for the following three impact conditions: PI, linear impact (LI), and drop impact (DI). The LI and PI conditions were performed with a HIII head form, while the DI condition was performed with both a HIII and NOCSAE head form. The HIII and NOCSAE head forms were developed by the University of Virginia, Center for Applied Biomechanics and were modeled with a deformable head skin and rigid skull.^21^ The DI conditions were modeled with a rigid neck, while the LI and PI conditions utilized the HIII head–neck with a deformable neck system. The PI followed protocol per the current STAR rating system developed at Virginia Tech^40^,^42^ and was modeled with a deformable nylon impacting surface, while the LI condition was modeled with a deformable nylon cap attached to a cylinder of vinyl nitrile foam.^21^ The DI condition was model as a rigid connection between the head form neck and a drop carriage and a drop plate modeled with a deformable pad on the surface of a rigid plate. Each loading condition was performed at varying impact locations with a set of prescribed velocities, which are shown in the Appendix (Tables A2, A3, A4, A5). The final test matrix consisted of 12 VT pendulum tests, 24 linear impactor tests, 19 DI tests with the NOCSAE head form, and 12 drop tower tests with the HIII head form. Each simulation was run for 40 ms and a total of 67 simulations were performed and are described in Table 3. The test data for the Schutt Air XP Pro was provided by Virginia Tech for the PIs, and Biomechanical Consulting and Research, LLC (Biocore) for the DIs and LIs. Details regarding each impact case can be found in Table 3 and a visual comparison of the test is shown in Fig. 3. Further details regarding the development and use of the impactor models can be seen in Giudice *et al*.^21^

The helmet model was validated through these impact conditions by comparing various kinematic and kinetic response to the physical experimental response, which included force, head linear acceleration, head angular velocity, and carriage acceleration (Table 3). A quantitative comparison of these measures was conducted using CORelation and Analysis (CORA),^19^,^49^ which is a widely used tool to validate computational FE models vs. experimental data. This study utilized the cross-correlation score which evaluates how similar two time-history curves match by comparing their phase, magnitude, and slope, and assigns a relative score from 0 to 1 where 1 represents a perfect match. This method was applied for any desired directional measure for all test cases. CORA scores were calculated using default parameters from time 0 to 30 ms. The corridor rating was not used. A detailed explanation for the mathematical calculation of CORA can be referenced in the CORA user manual (Thunert, 2012—Partnership for Dummy Technology and Biomechanics).^49^ Weighting factors based on experimental peak magnitude values were applied to calculate an average rating for a signal with orthogonal components. This weighting factor is referred to as the test magnitude factor, or TMF, which is calculated by normalizing the peak value for each orthogonal signal of a single sensor (e.g. the X, Y, and Z signals) by the sum of peaks for each orthogonal signal as per Eq. (1).^40^ Weighting was only applied to the orthogonal component signals from the same sensor.

1 $$ TMF = \frac{{R_{i} }}{{R_{x} + R_{y} + R_{z} }}. $$

The peak value of the test trace of a given signal is defined as *R*_*i*_ was applied to the orthogonal component signals from the same sensor. The magnitude factor is then applied to the CORA score for each respective orthogonal signal and the sum of these magnitude weighted signals yield a final CORA score for the sensor. An overall score for a given impact condition is obtained by averaging the scores of all test sensors. The overall score of helmet validation is the mean of all tests conducted.

## Results

### Material Characterization

The tension stress–strain response of thermoplastic outer shell was most comparable for the dynamic tests of 100 and 430/s, while the monotonic and cyclic quasi-static compression tests showed good peak response comparison but did not capture the relaxation phase of the experimental response (Fig. A1). Regarding the foam material characterization, monotonic compression response matched well for each of the foams and the foams themselves exhibit a stable response. The stability of these foams was considered a high priority as they would experience severe deformations and would be the most likely cause for simulation error terminations. During the cyclic quasi-static compression tests, the simulation matches the loading portion shape and peak response of the experimental data but did not completely capture the unloading portion of the test data (Fig. A3). Tensile response of the vinyl padding covering matched well between the simulation and experimental data in the 400/s rate and overestimated stiffness the quasi-static response. This is due to the lack of rate dependency for the *Mat_Fabric material model used for the vinyl padding. It was fit to the high rate tension data because it was closer to the rates that we would expect the material to undergo during an impact (Fig. A3).

Initial single cone compression results for the simulation (not shown) matched the experimental data but the full helmet response in all tests when donned with a head form underestimated the observed impact forces. Thus a tuned response was used to match the full helmet response. This overestimated both the dynamic (50 mm/s) and quasi-static (0.1 mm/s) rates (Fig. A5). This was justified because the cones were not tested at rates and deflection values approaching those experienced in full-helmet simulations (e.g. 3–10 m/s) based on test equipment limitations. While the single cone controlled testing here overestimated the structural response, the response begins to approach the data from quasi-static to dynamic rates. It’s possible that at higher rates and greater buckling it would continue to approach the true response. More on this specific point can be found in the Discussion. Chinstrap material response was very similar between the simulation and experiment up to 5% strain, which is similar to the maximum chinstrap strain that we observed in the full-helmet impacts. Plots of the material level validation for each component of the helmet model can be found in the Appendix.

### Sub-Assembly Component Testing

Compression response of the facemask was comparable between simulation and experiment for both the anterior–posterior and lateral compression (Fig. A8). The simulation underestimated stiffness of the thermoplastic outer shell for each direction of compression but the shape of the curves are similar. These component tests were only performed at quasi-static rates due to equipment restrictions. The complex nature of positioning the helmets and modeling the friction of the contact surfaces for these tests may explain this discrepancy (Fig. A9). The simulation response of both the open and closed foam component compression tests was similar to the experimental data in the loading regime. The open foam simulation results over-estimate peak force, while the closed foam is similar to the experiment. The unloading portion of the closed foam more closely represents the data than the open foam response. Both the open and closed foam components show rate dependency (Fig. A10). Plots of the component level validation for each component of the helmet model can be found in the Appendix.

### Full Helmet Response

The helmet model presented in this work is freely available *via* download along with the previously published head forms (see Acknowledgements). The total number of simulations run to develop and validate the helmet model, from material validation to full helmet impacts, amounted to 96 simulations. The helmet model was validated against a series of 67 experimental impacts including 12 VT pendulum tests, 24 linear impactor tests, 19 DI tests with the NOCSAE head form, and 12 drop tower tests with the HIII head form. Each of these full-helmet simulations normal terminated. Validation criteria included various outputs which varied between the test setups and can be seen in Table 3. Simulation kinematics and force outputs were generally comparable to experimental data for the varying test setups. A complete collection of trace comparisons may be found in the referenced data repository. Each helmet simulation was compared to the experiment quantitatively, with CORA, and qualitatively by trace comparison and visual time histories. This was to ensure that the resulting scores were achieved through expected and repeatable response of the helmet model. (i.e. that the model behaved similarly to the physical helmet during impact). An example of this process is shown in Table 4.

**Table 4:** Example comparison for each of the boundary condition setups for a single trace within the given boundary condition at a set impact speed and location.

	Trace comparison	Simulation	Experiment
LI: A 5.5 m/s			
NOCSAE DI: Front 6.0 m/s			
HIII DI: Front 4.9 m/s			
PI: Side 6.1 m/s			NA

The CORA score provided was calculated from the provided trace. A visual comparison of the model and experiment is shown as well. Note that there was no recorded video for the pendulum impactor experiments

*NA* video was not available for comparison from the physical pendulum impact tests

Overall CORA scores and standard deviations for each impact condition are shown in Table 5. Total CORA scores ranged from 0.7 to 0.8, which is analogous to other helmet models^5^ or FE validation studies in general.^10^,^11^,^21^,^38^,^50^ Scores for individual output traces ranged from 0.5 to 0.95. Head kinematics of the simulations generally followed the shape of the experimental traces, even with more complex bi-modal responses. Peak forces aligned well in most simulations, but varied in accuracy within certain test setups, with the HIII DIs showing the most variability. Carriage accelerations showed high levels of agreement. Further details on CORA and the calculation of these values can be found in the methods section. A complete breakdown of the CORA results including all the impact locations and speeds, as well as scores for the individual outputs, can be found in the Appendix.

**Table 5:** Overall CORA score averages and standard deviations for all of the simulations within each impact condition.

	Drop tower NOCSAE	Drop tower HIII	Linear impactor	Pendulum
Overall weighted CORA score	0.785 ± 0.081	0.709 ± 0.09	0.779 ± 0.049	0.781 ± 0.102

## Discussion

A FE model of an American football helmet was developed and validated through a rigorous series of impact conditions, including material and component level validation. Physical measurements such as size, shape, and mass of the individual components, along with the fully assembled helmet, were within reasonable tolerances. The model has shown the ability to obtain kinematic and force response comparable to experimental data through many different directional loading scenarios. Given existing ATD and human body models, and established injury criteria based on acceleration or strain, this model can be viewed as another tool for engineers and biomechanists to advance head injury research.

The two different ATD head forms did not have a definitive impact on comparisons. The drop tests with the NOCSAE head showed better correlation than the HIII head, while the linear impactor and pendulum tests both used the HIII head and showed similar scores to the NOCSAE head drop tests. Impact response had some degree of sensitivity to the position of the helmet on the head form and the same helmet fit was used for all HIII test cases. Slight differences in the position of the helmet between experimental test setups and individual loading scenarios may have some influence in the differences seen in the response comparison. Within the NOCSAE head form DI test matrix, the back and facemask impacts were used to test robustness and were not used for CORA evaluation. This is due to excessive rotation in the nominally rigid connection during testing, which could not be captured in the model. Additionally, the NOCSAE head form facemask drop was only tested at 2.9, 3.7, and 4.9 m/s.

There were some limitations during the modeling and validation process. In the physical helmet, the foam for the helmet pads are free-floating within the grey vinyl. The modeling approach of meshing the 2D vinyl along coincident nodes of the 3D foam was chosen to minimize contacts and maximize the stability of the model. Material testing data for the vinyl foam covering was gathered and an initial hyperelastic material model was developed. However, in order to use this covering to model the air component of these pads using airbags, the vinyl was assigned a fabric material.

The force response of the helmet impacts appeared to rely heavily on the buckling properties of the conical absorbers, which were seen to not plastically deform when fully compressed in physical testing. This buckling response appeared during impacts a 5 m/s and above generally, which encompasses over half of the simulated impacts. The cone material characterization test did not reach this level of deformation, and thus did not capture this buckling behavior, so we were unable to compare the response of the cones post-buckling between the simulation and experimental data. In the Appendix, we have provided a visual comparison of the cone buckling between the single cone compression test and two full-helmet impacts (Fig. A6). We have also provided stress heat map images for the components of the helmet during an impact (Fig. A12). While a single cone was tested, in the helmet design there are several unique anatomical features of the conical absorbers (e.g. interconnects and variable cones sizes) which presented a challenge to develop a characteristic material model. A constitutive model that fit the cone characterization test well, provided much too soft of a macro response in the full helmet impacts. Therefore, the material of the cones was chosen that would not plastically deform, capture reasonable pre-buckling behavior in the cone compression tests, and retain enough stiffness post-buckling to achieve realistic response in full-helmet impacts. We decided that a tuned response of a complex geometry was better than a fundamentally flawed material response (plastic deformation after impact). It should also be noted that the conical absorber component of the helmet is composed of over a hundred individual cones, most of which are not aligned perfectly normal to the shell of the helmet. This yields a much more complex system for loading than a single cone axial compression test. Off-axis and shear testing of the cones would have been useful data to validate with the simulation but was not performed due to equipment limitations.

During the simulation, the helmet model had 3% added mass total, 55% of which came from the conical absorbers, equating an 8% increase in the conical absorbers themselves. These are tolerable values and are to be expected when the conical absorbers have the smallest timestep and the most elements of any part in the helmet.

There were two cases where the time interval for CORA was altered from the first 30 to 20 ms. The experimental traces of the 6.1 m/s side and back PIs displayed a noisy increase in carriage acceleration from 20 to 30 ms. The behavior of the helmet during this noise could not be determined due to the lack of video recordings. However, it occurs after the impact and was determined to be reasonable to exclude from the CORA evaluation. Regarding the linear impactor frontal impacts, video from the physical test showed that the nylon cap–vinyl nitrile foam interface separated from the impactor after impact with the helmet. This behavior was not possible in the simulations as these components were fixed together. This could influence the results since the impactor may restrict the helmet/head motion in FE simulation. Also, note that the 1D component of the chinstrap is defined as an elastic spring discrete beam and has a default initial force of 40 N. This is to ensure that the chinstrap is snug with the head form prior to impact. Environmental factors such as temperature and humidity were not considered during model development. It should be noted that a goal of the project was to be able to simulate a helmet impact on a personal computer within a reasonable amount of time. We found that a 30 ms simulation with this helmet model could run in approximately 3 h on a desktop workstation (Intel Core i7-4790K, 16 GB memory), however, performance will vary among computers.

Head injuries in contact sports, particularly American football occur at relatively high rates in comparisons to other sports and helmets are the player’s primary source of head protection.^6^,^40^,^42^ FE computational models provide a tool for researchers to study and potentially optimize helmet designs for better player protection. This FE helmet model can be used in conjunction with ATD and human body models to study the relationship between helmet characteristics and head injury prevalence. This can help streamline the process of designing and testing helmets, which in turn may help reduce cost and improve helmet performance. The modeling processes shown here may also help influence the development of helmets in other applications such as other sports and motorsports.

In conclusion, the model described in this study has been extensively validated and can function as a building block for innovation in player safety. These publicly available FE models will function as a platform and baseline resource for injury prevention research in order to stimulate the development of novel, effective helmet designs.

## Electronic supplementary material

Below is the link to the electronic supplementary material.

Supplementary material 1 (PDF 1451 kb). Additional data which includes time-history plots for each full-helmet impact are available from FigShare https://figshare.com/projects/Development_and_Multi-Scale_Validation_of_Finite_Element_Football_Helmet_Model/62522
