# Supplementary material for: Development and Multi-Scale Validation of a Finite Element Football Helmet Model
Source: Ann Biomed Eng. 2019 Sep 13;48(1):258–70. doi: 10.1007/s10439-019-02345-7 (PMC6928099; doi:10.1007/s10439-019-02345-7)
Supplement: Supplementary file 1 — Supplementary material 1 (PDF 1451 kb). Additional data which includes time-history plots for each full-helmet impact are available from FigShare https://figshare.com/projects/Development_and_Multi-Scale_Validation_of_Finite_Element_Football_Helmet_Model/62522 [file 10439_2019_2345_MOESM1_ESM.pdf]

Appendix: 2016 Schutt Air XP Pro v.1.0

Figures 2

Tables 2

Physical Measurement Comparison 4

Material Testing Validation 5

Component Testing Validation 11

Validation Test Overview 13

Correlation Analysis 19

Figures

Figure A1. Material testing validation for the thermoplastic outer shell. 5

Figure A2. Visualizes the position of each type of foam tested relative to the helmet. 6

Figure A3. Material testing validation for the foam. 8

Figure A4. Material testing validation for the vinyl padding covering. 8

Figure A5. Material testing validation for the conical absorbers. 9

Figure A6. Shows varying degrees of conical absorber buckling between the single cone compression test and the full-helmet 4.9 m/s frontal Hybrid III drop test. A) Single cone compression test at max deflection B) Compression of the conical absorbers at the start of the simulation C) Compression of the conical absorbers at 10 ms. Note that parts of the helmet have been visually hidden to highlight the conical absorber response. 9

Figure A7. Material testing validation for the chinstrap. 10

Figure A8. Component level testing validation for the facemask. 11

Figure A9. Component level testing validation for the thermoplastic outer shell. The data begins after an initial preloading. 11

Figure A10. Component level testing validation for the meso-scale padding. High rate 10 m/s filtered 10,000 Hz SAE ms. 12

Figure A11. Fitting of helmet onto a HIII head form. 17

Figure A12. Visual heat map of first principle stress experienced by A) Thermoplastic outer shell, B) Conical absorbers, C) Foam, D) Chinstrap in the 6.1 m/s front boss pendulum impact. 17

Figure A13. Histogram showing the distribution of CORA scores for each boundary condition setup by number of simulations for each range of CORA scores. 22

Tables

Table A1. Physical thickness of model components and a mass comparison between the model and physical helmet. 4

Table A2. Linear impactor (LI) validation tests. 13

Table A3. Drop tower (DI) validation tests with NOCSAE head form. 14

Table A4. Drop tower (DI) validation tests with HIII head form. 15

Table A5. Pendulum impact (PI) validation tests. 16

Table A6. Breakdown of the various helmet components in terms of element type, size, and total element number. 18

Table A7. CORA scores for the pendulum impact (PI) with hybrid III head form. 19

Table A8. CORA scores for the linear impact (LI) with hybrid III head form. 20

Table A9. CORA scores for the drop impact (DI) with NOCSAE head form. 21

Table A10. CORA scores for the drop impact (DI) with Hybrid III head form. 21

Physical Measurement Comparison

Table A1. Physical thickness of model components and a mass comparison between the model and physical helmet.

Component	Thickness (mm)	Measured Mass (kg)	Model Mass (kg)
Facemask	5.19 / 6.15	0.451	0.452
Outer Shell	3.55	0.673	0.673
Conical Absorbers	1.09	0.281	0.292
Vinyl Foam Covering	0.50 / 0.60	0.203	0.26
Foams	7.5 – 11.9		
Chin Cup/Strap	2.00	0.062	0.035
**Total**	**~**	**1.670**	**1.712**

Material Testing Validation

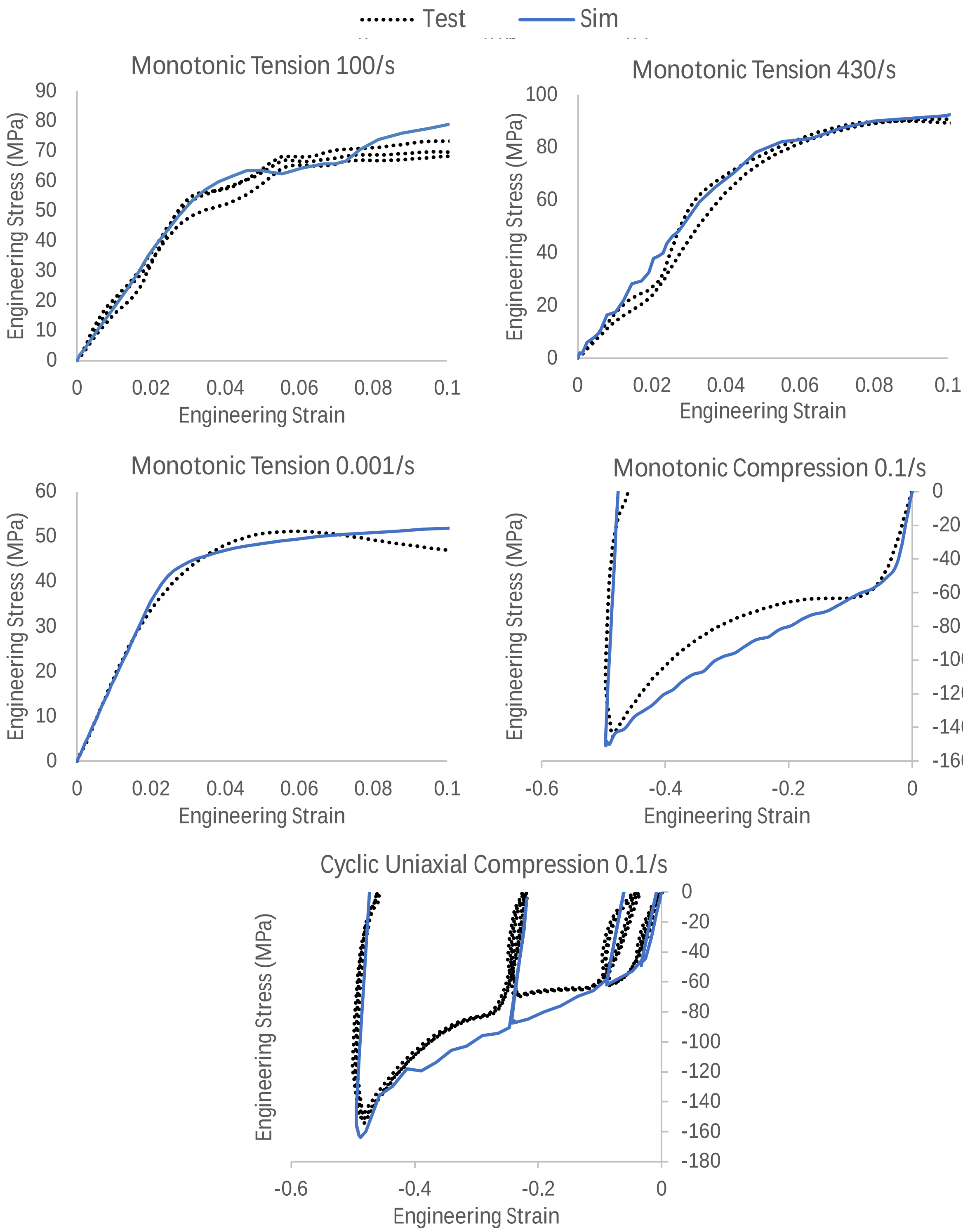

Figure A1. Material testing validation for the thermoplastic outer shell.

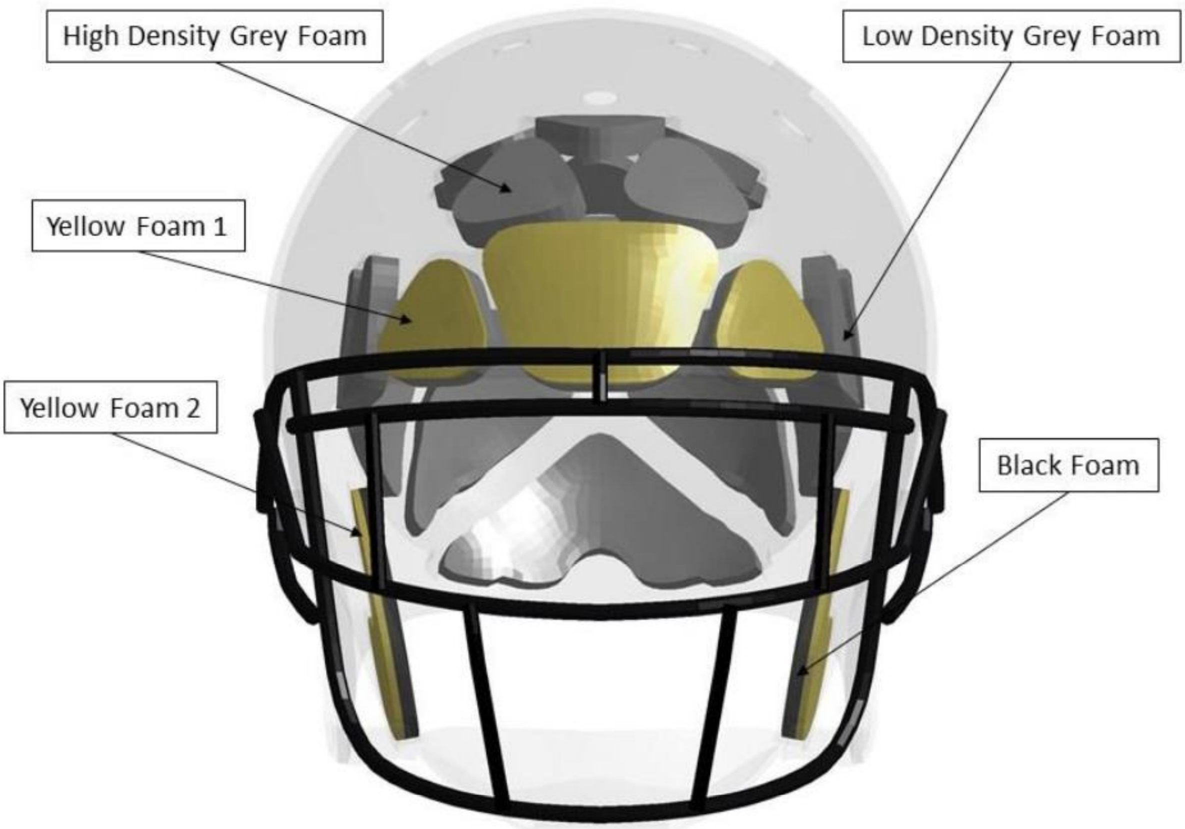

Figure A2. Visualizes the position of each type of foam tested relative to the helmet.

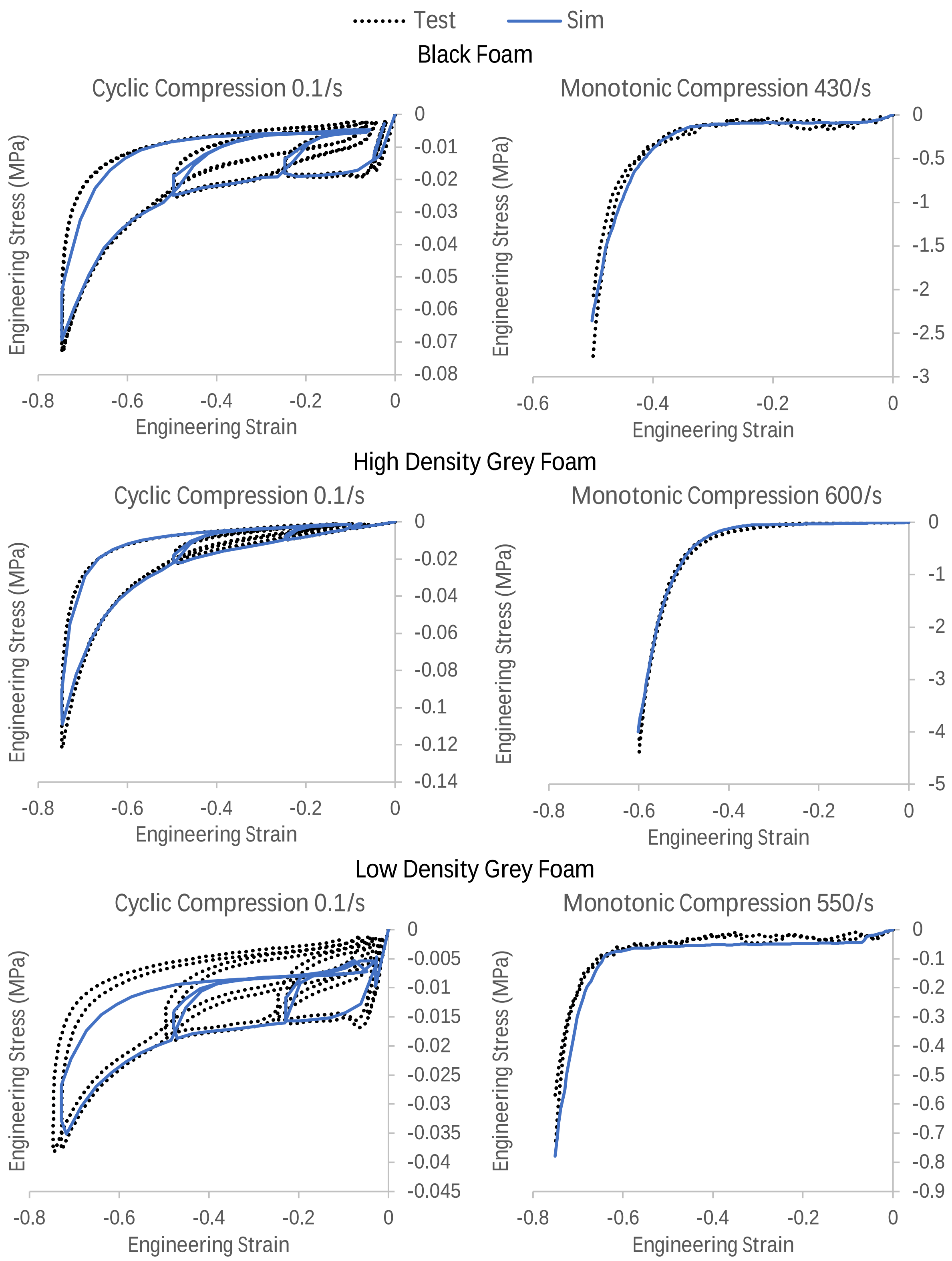
Test
Sim
Black Foam
Cyclic Compression 0.1/s
Monotonic Compression 430/s
High Density Grey Foam
Cyclic Compression 0.1/s
Monotonic Compression 600/s
Low Density Grey Foam
Cyclic Compression 0.1/s
Monotonic Compression 550/s
Engineering Stress (MPa)
Engineering Strain

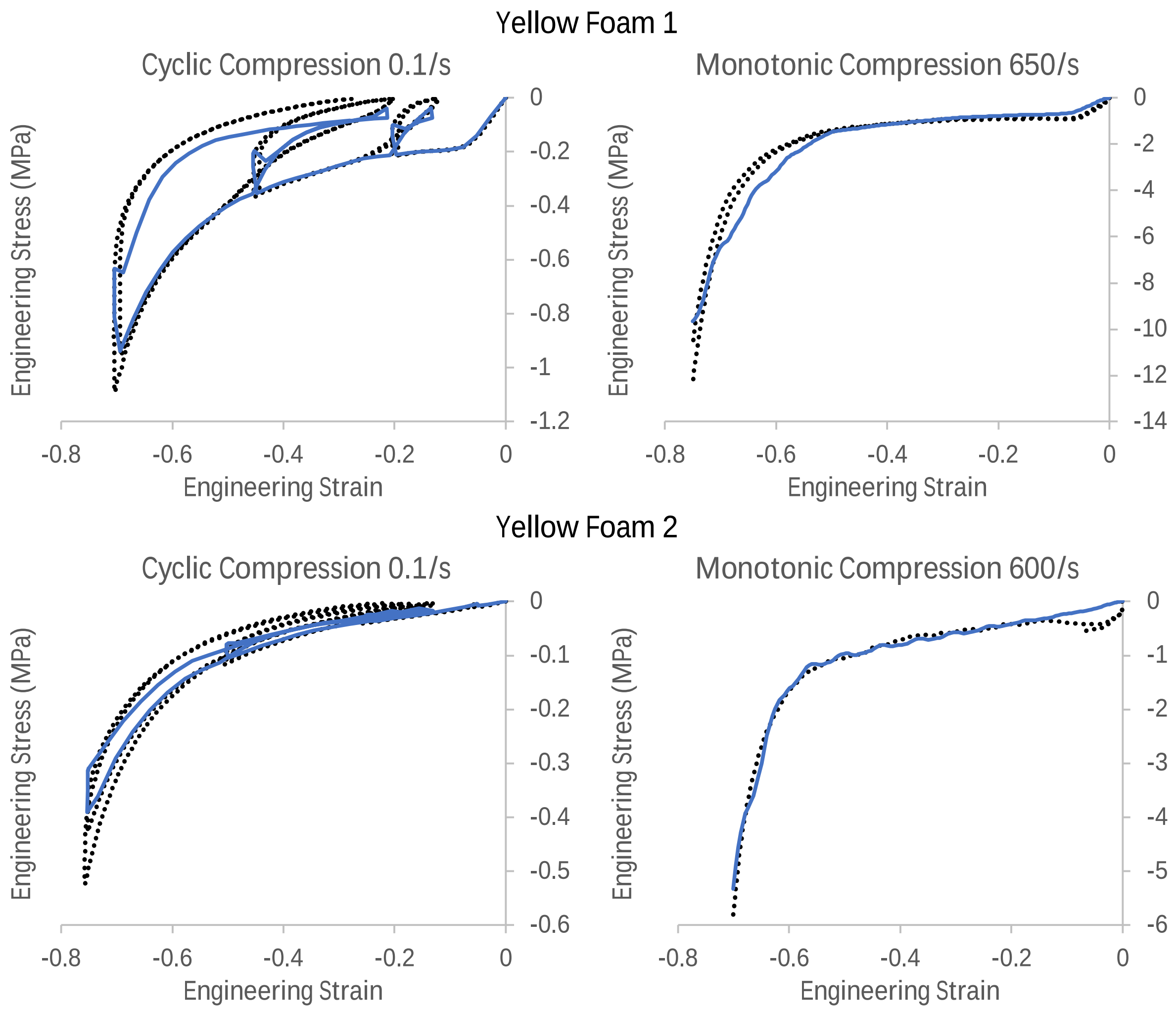

Figure A3. Material testing validation for the foam.

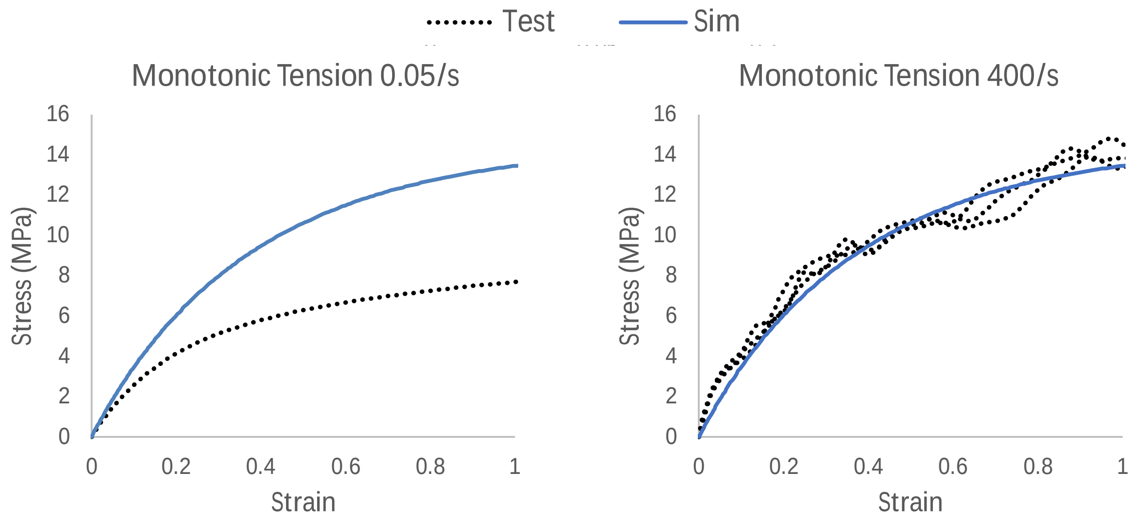

Figure A4. Material testing validation for the vinyl padding covering.

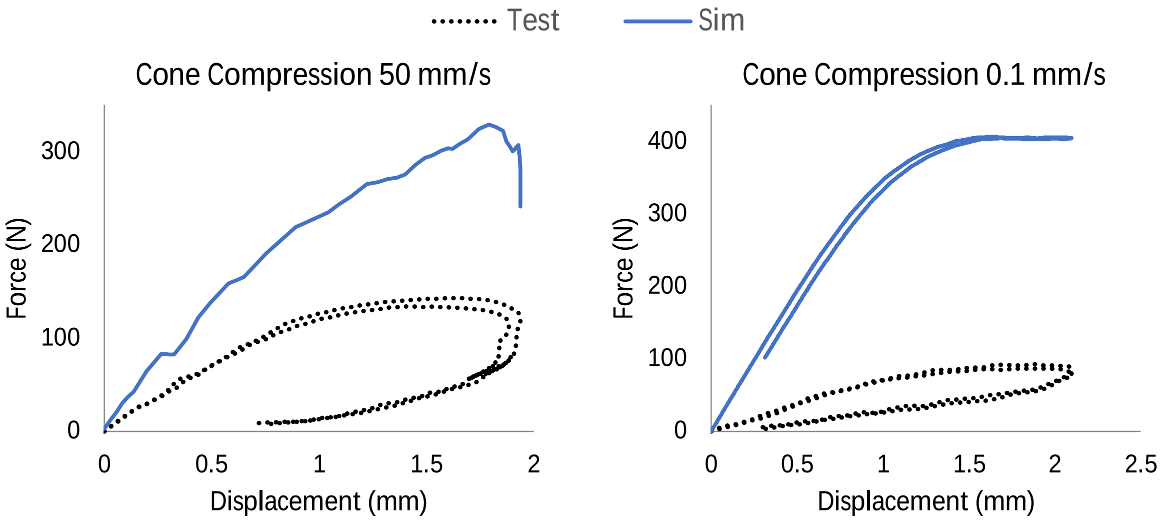

Figure A5. Material testing validation for the conical absorbers.

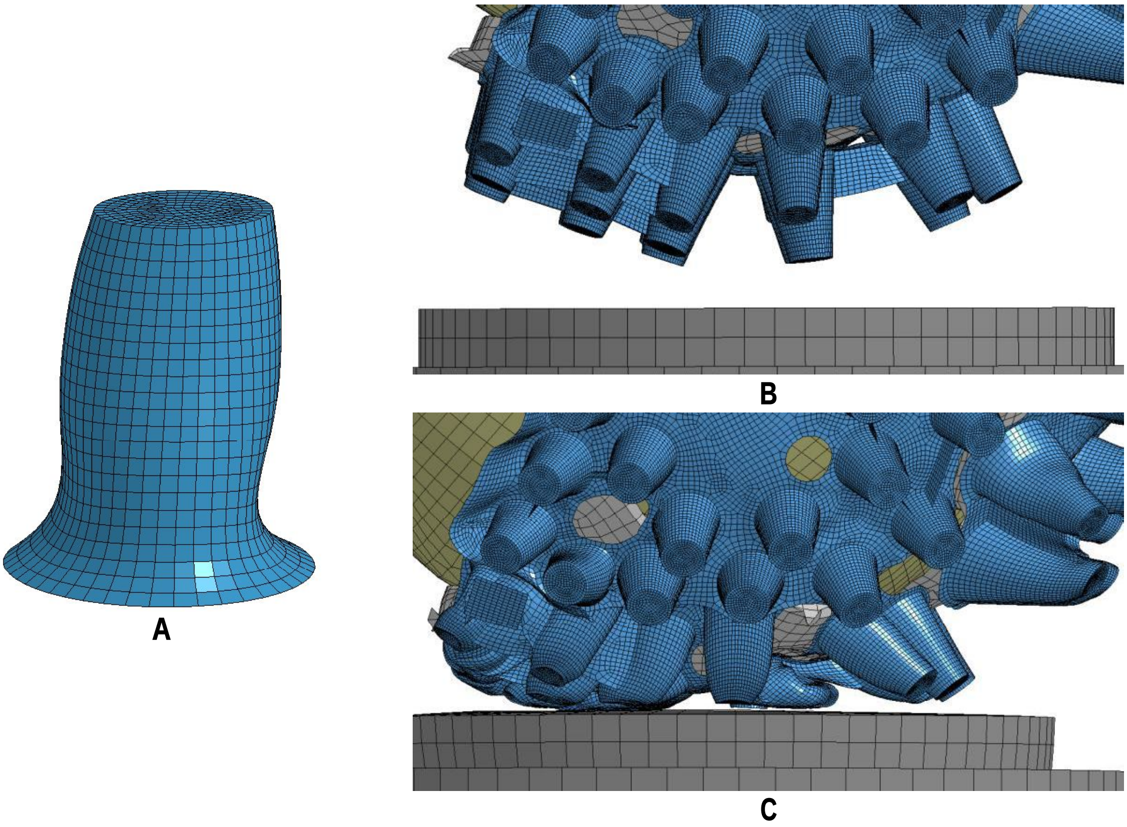

Figure A6. Shows varying degrees of conical absorber buckling between the single cone compression test and the full-helmet 4.9 m/s frontal Hybrid III drop test. A) Single cone compression test at max deflection B) Compression of the conical absorbers at the start of the simulation C) Compression of the conical absorbers at 10 ms. Note that parts of the helmet have been visually hidden to highlight the conical absorber response.

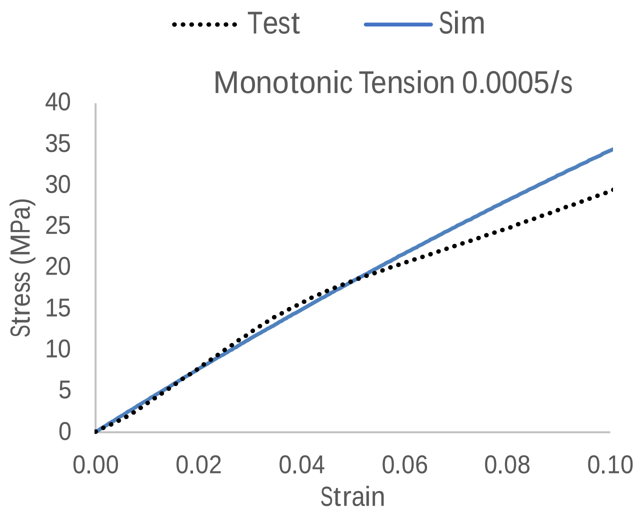

Figure A7. Material testing validation for the chinstrap.

Component Testing Validation

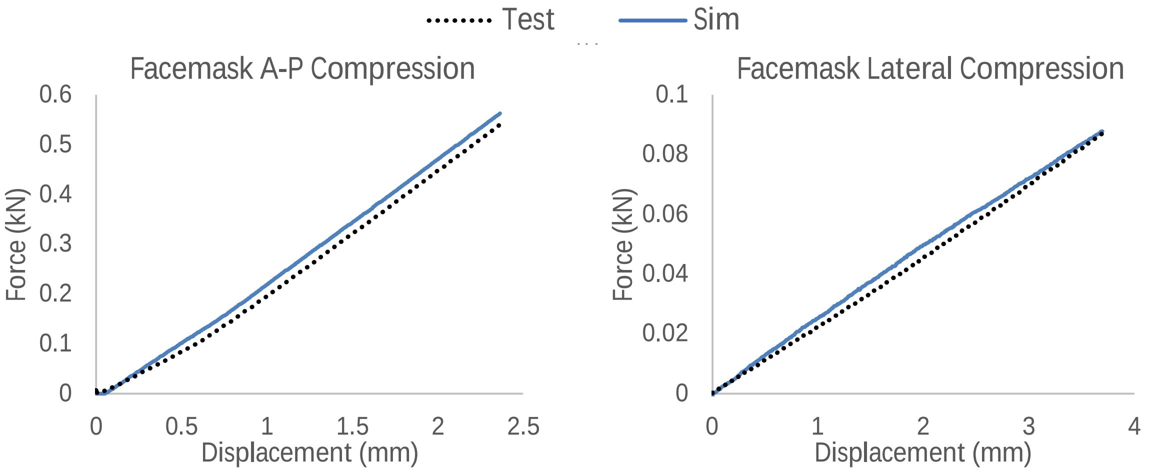

Figure A8. Component level testing validation for the facemask.

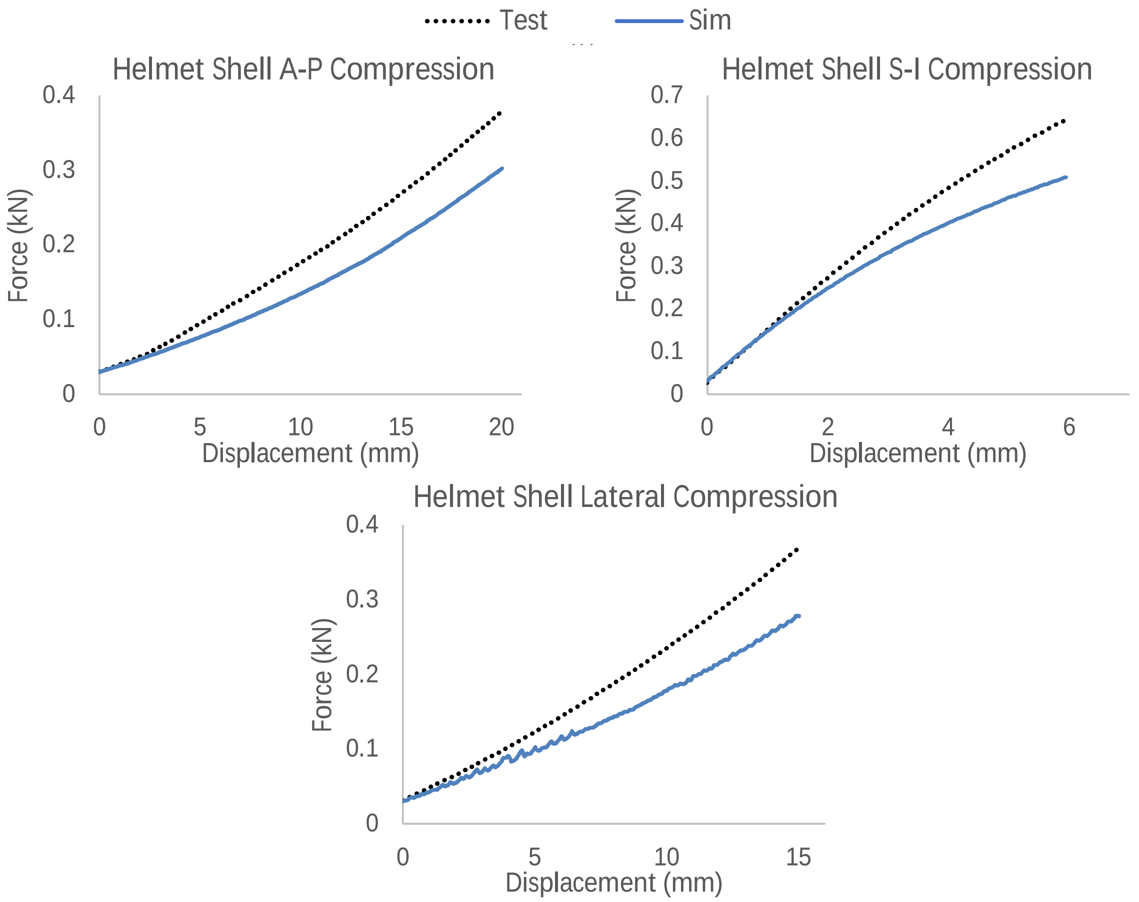

Figure A9. Component level testing validation for the thermoplastic outer shell. The data begins after an initial preloading.

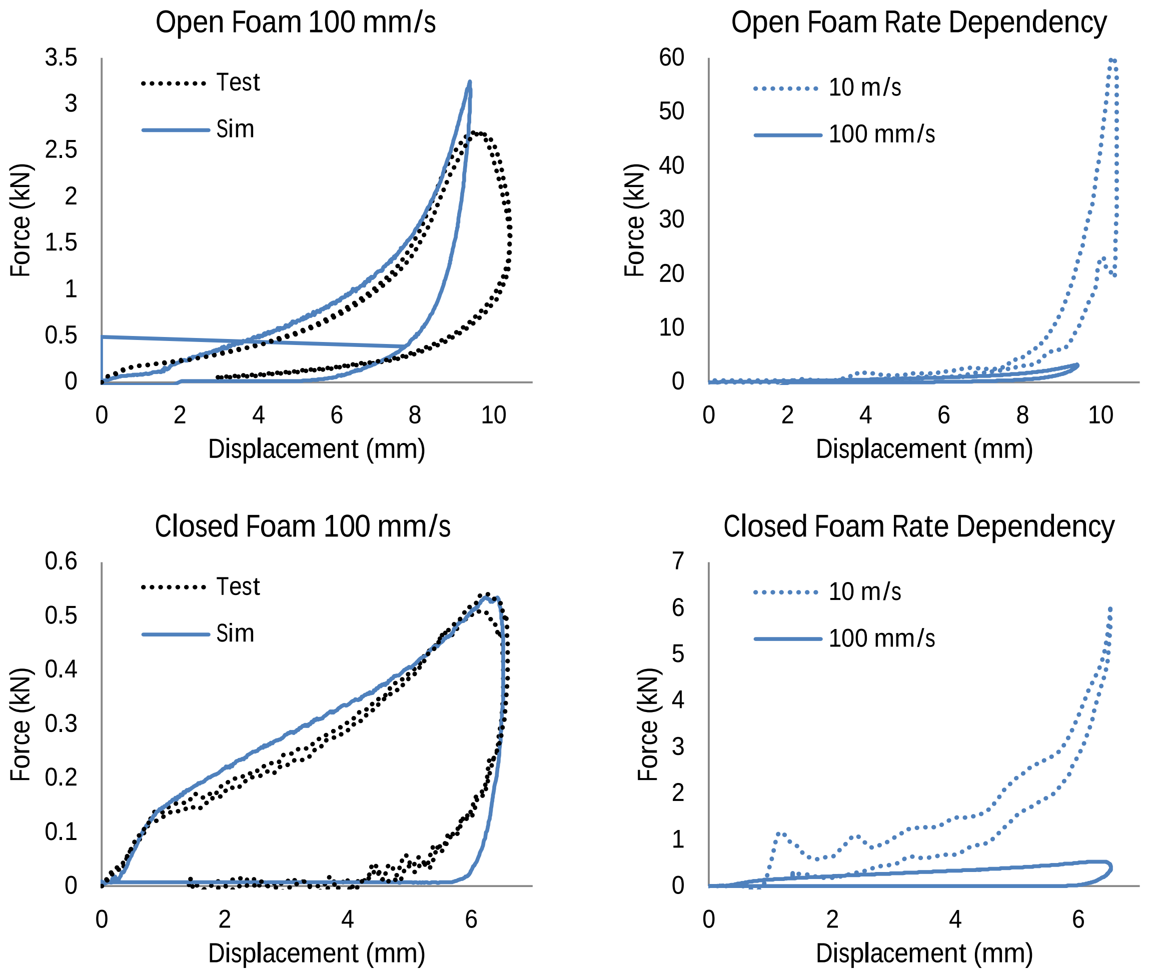

Figure A10. Component level testing validation for the meso-scale padding. High rate 10 m/s filtered 10,000 Hz SAE ms.

Validation Test Overview

Table A2. Linear impactor (LI) validation tests.

Impact Configuration		Evaluation Criteria				Simulation	Experiment
	Impact Velocity [m/s]	Force versus Time	Linear Acceleration versus Time		Angular Velocity		
**A**	5.5 7.4 9.3	Contact Force (Impact direction)	Head CG (XYZ)	Impactor (Impact direction)	Head CG (XYZ)	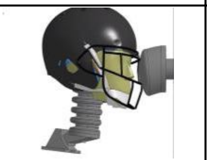	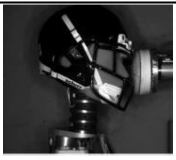
**AP**	5.5 7.4 9.3	Contact Force (Impact direction)	Head CG (XYZ)	Impactor (Impact direction)	Head CG (XYZ)	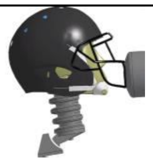	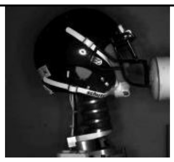
**B**	5.5 7.4 9.3	Contact Force (Impact direction)	Head CG (XYZ)	Impactor (Impact direction)	Head CG (XYZ)	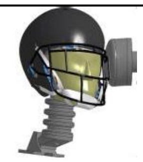	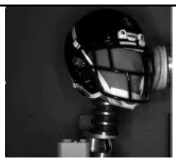
**C**	5.5 7.4 9.3	Contact Force (Impact direction)	Head CG (XYZ)	Impactor (Impact direction)	Head CG (XYZ)	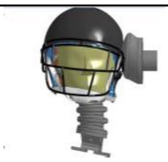	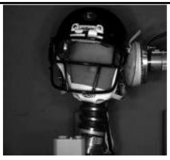
**D**	5.5 7.4 9.3	Contact Force (Impact direction)	Head CG (XYZ)	Impactor (Impact direction)	Head CG (XYZ)	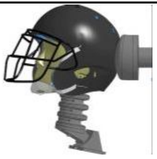	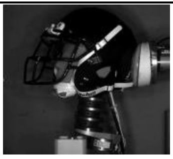
**F**	5.5 7.4 9.3	Contact Force (Impact direction)	Head CG (XYZ)	Impactor (Impact direction)	Head CG (XYZ)	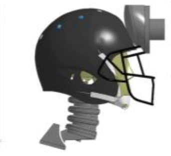	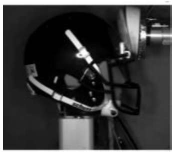
**R**	5.5 7.4 9.3	Contact Force (Impact direction)	Head CG (XYZ)	Impactor (Impact direction)	Head CG (XYZ)	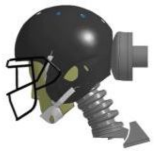	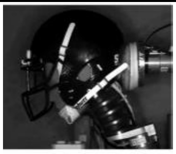
**UT**	5.5 7.4 9.3	Contact Force (Impact direction)	Head CG (XYZ)	Impactor (Impact direction)	Head CG (XYZ)	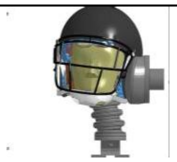	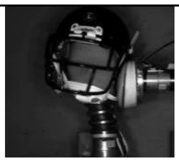

Table A3. Drop tower (DI) validation tests with NOCSAE head form.

Impact Configuration		Evaluation Criteria			Simulation	Experiment
	Drop Height [ft]	Force versus Time	Linear Acceleration versus Time			
**Back***	1.6<br>2.6<br>4.6<br>7.0	Contact Force (XZ)	Head CG (X)	Carriage Acc. Z	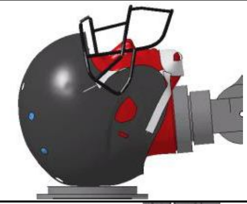	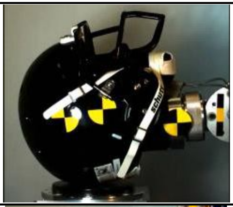
**Front**	1.6<br>2.6<br>4.6<br>7.0	Contact Force (XZ)	Head CG (XZ)	Carriage Acc. Z	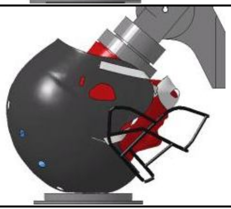	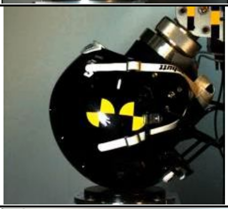
**Mask***	1.6<br>2.6<br>4.6	Contact Force (XZ)	Head CG (X)	Carriage Acc. Z	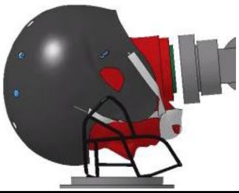	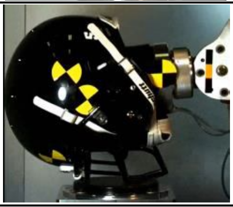
**Side**	1.6<br>2.6<br>4.6<br>7.0	Contact Force (XZ)	Head CG (YZ)	Carriage Acc. Z	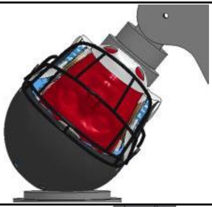	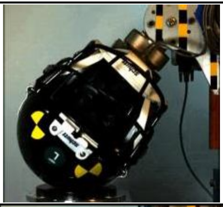
**Top**	1.6<br>2.6<br>4.6<br>7.0	Contact Force (XZ)	Head CG (XZ)	Carriage Acc. Z	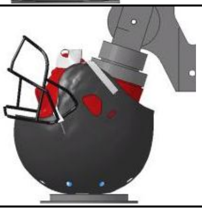	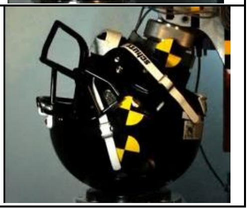

Table A4. Drop tower (DI) validation tests with HIII head form.

Impact Configuration		Evaluation Criteria			Simulation	Experiment
	Drop Height [ft]	Force versus Time	Linear Acceleration versus Time			
**Back**	1.6<br>4.6<br>7.0	Contact Force (XZ)	Head CG (XYZ)	Carriage Acc. Z	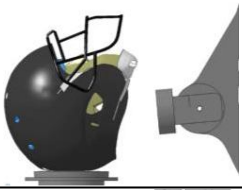	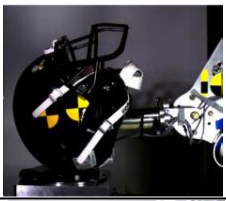
**Front**	1.6<br>4.6<br>7.0	Contact Force (XZ)	Head CG (XYZ)	Carriage Acc. Z	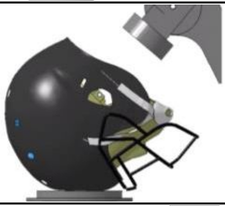	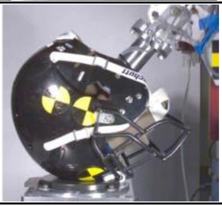
**Side**	1.6<br>4.6<br>7.0	Contact Force (XZ)	Head CG (XYZ)	Carriage Acc. Z	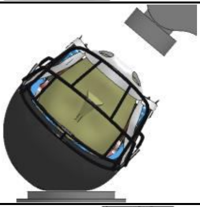	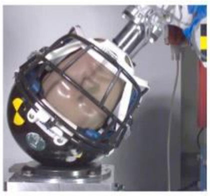
**Top**	1.6<br>4.6<br>7.0	Contact Force (XZ)	Head CG (XYZ)	Carriage Acc. Z	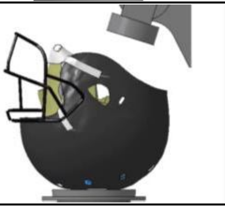	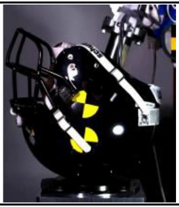

Table A5. Pendulum impact (PI) validation tests.

Impact Configuration		Evaluation Criteria				Simulation
	Impact Velocity [m/s]	Force versus Time	Linear Acceleration versus Time		Angular Velocity	
**Front**	3.0	Contact Force (Impact direction)	Head CG (XYZ)	Impactor (Impact direction)	Head CG (XYZ)	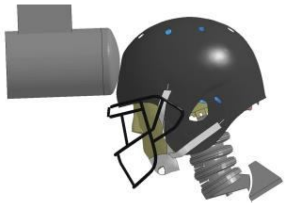
	4.6					
	6.1					
**Front Boss**	3.0	Contact Force (Impact direction)	Head CG (XYZ)	Impactor (Impact direction)	Head CG (XYZ)	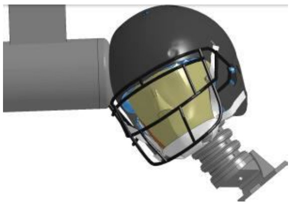
	4.6					
	6.1					
**Side**	3.0	Contact Force (Impact direction)	Head CG (XYZ)	Impactor (Impact direction)	Head CG (XYZ)	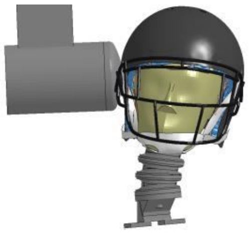
	4.6					
	6.1					
**Back**	3.0	Contact Force (Impact direction)	Head CG (XYZ)	Impactor (Impact direction)	Head CG (XYZ)	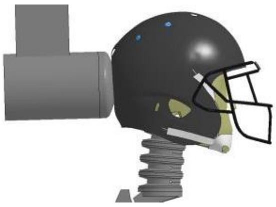
	4.6					
	6.1					

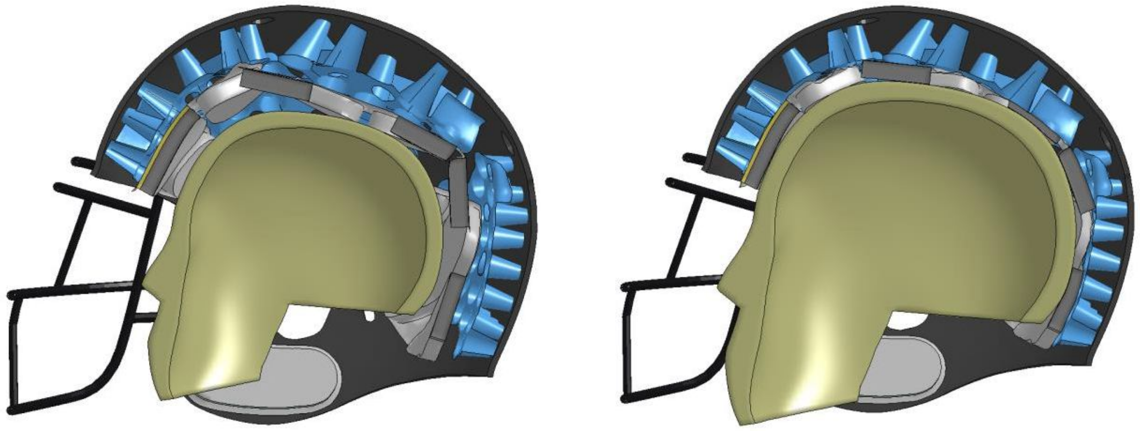

Figure A11. Fitting of helmet onto a HIII head form.

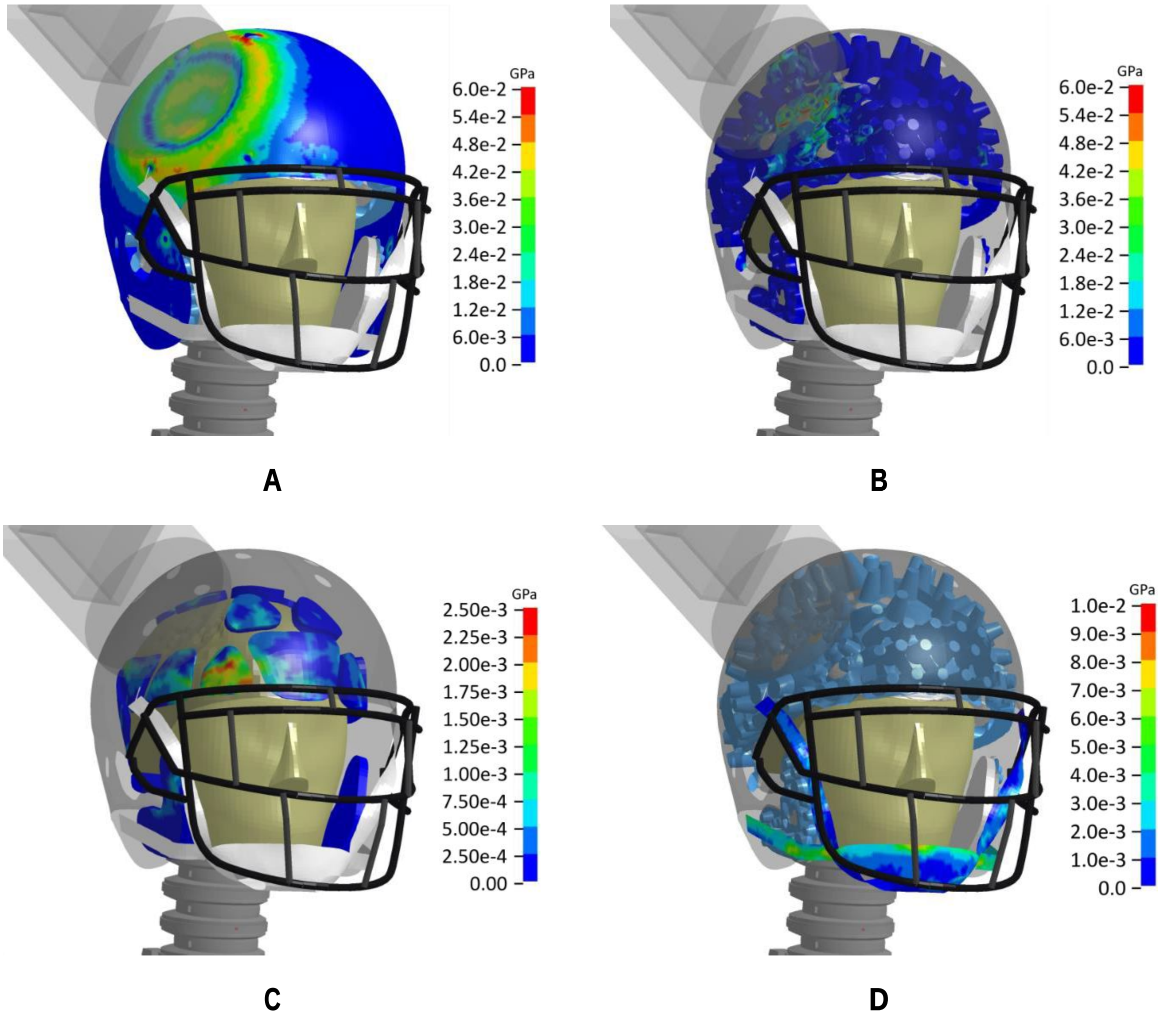

Figure A12. Visual heat map of first principle stress experienced by A) Thermoplastic outer shell, B) Conical absorbers, C) Foam, D) Chinstrap in the 6.1 m/s front boss pendulum impact.

Table A6. Breakdown of the various helmet components in terms of element type, size, and total element number.

Component	Element Type	Element Edge Length (Approx.)	# Elements
Facemask	1D Beam	10 mm	202
Helmet Shell	2D Quad	3 mm	22,418
Conical Absorbers	2D Quad	1 mm	225,673
Vinyl Covering	2D Quad	3.5 mm	12,926
Foams	3D Hexahedral	3 – 3.5 mm	13,284
Chin Cup	2D Quad	3 mm	780
Chin Strap	2D Quad / 1D Beam	3.1 mm / single beam	870 / 4
**Full Helmet**			**276,157**

*Vinyl covering shares coincident nodes with underlying foam.

Correlation Analysis

Table A7. CORA scores for the pendulum impact (PI) with hybrid III head form.

Pendulum Impact (PI)				
CORA	Carriage_Accel	Accel	Ang_Vel	Total
Front_1	0.821	0.756	0.543	0.706
Front_2	0.629	0.695	0.647	0.657
Front_3	0.535	0.649	0.646	0.610
Front_Boss_1	0.758	0.582	0.720	0.687
Front_Boss_2	0.778	0.645	0.833	0.752
Front_Boss_3	0.662	0.603	0.753	0.672
Back_1	0.804	0.822	0.836	0.820
Back_2	0.946	0.894	0.868	0.902
Back_3	0.860	0.780	0.894	0.845
Side_1	0.839	0.815	0.865	0.840
Side_2	0.922	0.893	0.906	0.907
Side_3	0.926	0.871	0.904	0.900
				**0.775**

Table A8. CORA scores for the linear impact (LI) with hybrid III head form.

Linear Impact (LI)					
CORA	Carriage_Accel	Head_Lin_Accel	Head_Ang_Vel	Force	Total
AP_5	0.862	0.741	0.707	0.726	0.759
AP_7	0.757	0.721	0.832	0.755	0.766
AP_9	0.882	0.793	0.832	0.852	0.840
A_5	0.864	0.805	0.676	0.792	0.784
A_7	0.917	0.761	0.720	0.816	0.804
A_9	0.567	0.775	0.689	0.777	0.702
B_5	0.930	0.866	0.821	0.719	0.834
B_7	0.917	0.865	0.874	0.741	0.850
B_9	0.933	0.862	0.881	0.787	0.866
C_5	0.871	0.835	0.740	0.866	0.828
C_7	0.869	0.818	0.738	0.822	0.812
C_9	0.872	0.812	0.724	0.825	0.808
D_5	0.825	0.779	0.896	0.665	0.791
D_7	0.831	0.784	0.905	0.655	0.794
D_9	0.728	0.789	0.875	0.663	0.764
F_5	0.645	0.673	0.641	0.728	0.672
F_7	0.838	0.671	0.668	0.783	0.740
F_9	0.649	0.796	0.724	0.676	0.711
R_5	0.865	0.804	0.687	0.772	0.782
R_7	0.861	0.791	0.738	0.780	0.793
R_9	0.825	0.813	0.790	0.812	0.810
UT_5	0.750	0.752	0.821	0.679	0.750
UT_7	0.766	0.769	0.760	0.660	0.739
UT_9	0.588	0.768	0.769	0.694	0.705
					**0.779**

Table A9. CORA scores for the drop impact (DI) with NOCSAE head form.

Drop Impact NOCSAE (DI)				
CORA	Carriage_Accel	Accel	Force	Total
Front_1	0.980	0.820	0.838	0.879
Front_2	0.899	0.829	0.789	0.839
Front_3	0.854	0.850	0.798	0.834
Front_4	0.882	0.805	0.827	0.838
Top_1	0.753	0.531	0.712	0.665
Top_2	0.689	0.626	0.508	0.608
Top_3	0.816	0.633	0.711	0.720
Top_4	0.872	0.618	0.789	0.759
Side_1	0.889	0.567	0.810	0.755
Side_2	0.956	0.719	0.871	0.848
Side_3	0.973	0.702	0.906	0.860
Side_4	0.954	0.625	0.864	0.814
				**0.785**

Table A10. CORA scores for the drop impact (DI) with Hybrid III head form.

Drop Impact Hybrid III (DI)				
CORA	Carriage_Accel	Accel	Force	Total
Front_1	0.851	0.808	0.805	0.821
Front_3	0.897	0.837	0.911	0.882
Front_4	0.911	0.774	0.885	0.857
Back_1	0.808	0.578	0.689	0.692
Back_3	0.752	0.648	0.680	0.693
Back_4	0.691	0.669	0.728	0.696
Top_1	0.665	0.539	0.551	0.585
Top_3	0.790	0.600	0.597	0.662
Top_4	0.697	0.573	0.654	0.641
Side_1	0.682	0.588	0.614	0.628
Side_3	0.798	0.576	0.725	0.699
Side_4	0.726	0.534	0.704	0.654
				**0.709**

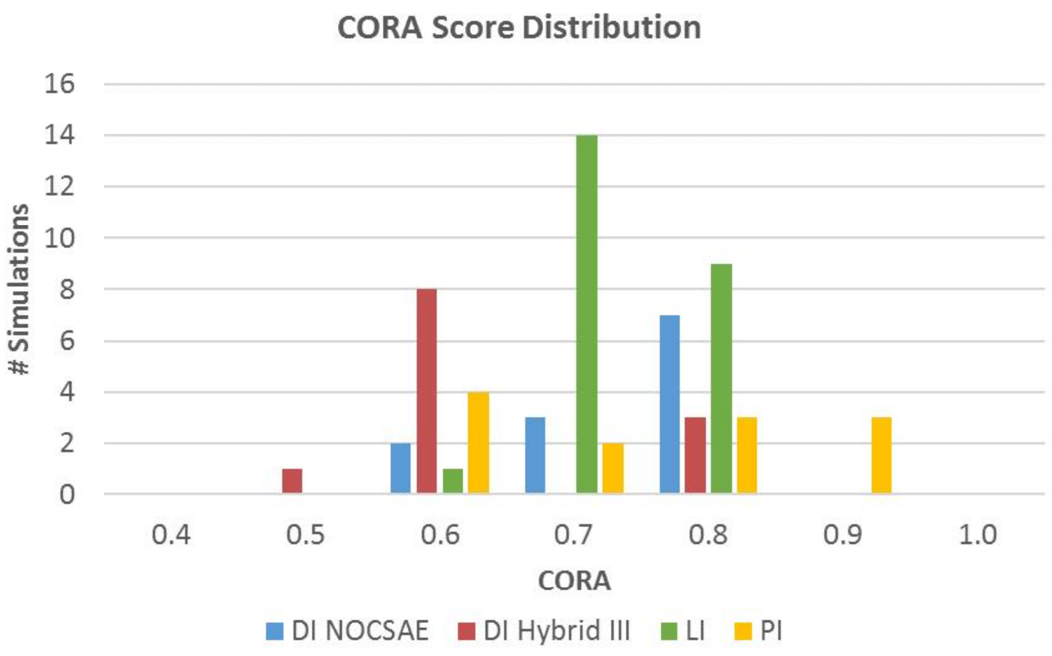

Figure A13. Histogram showing the distribution of CORA scores for each boundary condition setup by number of simulations for each range of CORA scores.
